# Navigating the Diagnosis and Treatment of Astroblastoma: A Pediatric Case Report

**DOI:** 10.7759/cureus.54901

**Published:** 2024-02-25

**Authors:** Haysum Khan, Mahrukh Afreen, Abdullah Khan, Saif Ali Malik, Mohammad Imran Bhatti

**Affiliations:** 1 Neurological Surgery, Shifa College of Medicine, Shifa Tameer-e-Millat University, Islamabad, PAK; 2 Neurosurgery, Shifa International Hospital Islamabad, Islamabad, PAK; 3 Internal Medicine, Shifa College of Medicine, Shifa Tameer-e-Millat University, Islamabad, PAK

**Keywords:** glial tumor, histogenesis, low grade astroblastoma, high grade astroblastoma, astroblastoma

## Abstract

Astroblastoma, a rare glial tumor of the central nervous system, presents diagnostic and therapeutic challenges due to its low incidence and variable clinical presentations. In this case study, we present the case of an 11-year-old boy with high-grade astroblastoma, highlighting the complexities in diagnosis and treatment. The clinical presentation initially involved right-sided motor weakness, which, after undergoing a brain MRI, revealed a large solid cystic mass in the left parietal lobe. Histopathological examination after undergoing surgery confirmed an astroblastoma with high-grade features, characterized by increased cellularity and high mitotic activity. Immunostaining patterns supported the glial origin of the tumor. Gross total resection remains the primary approach for its treatment, but adjuvant therapies for high-grade astroblastomas are still evolving, offering potential life-changing possibilities for the future. Due to its rarity, collecting sufficient data to develop an effective treatment protocol for this uncommon tumor is very challenging. This case underscores the importance of combined efforts and ongoing research to effectively navigate the diagnosis and treatment of astroblastoma.

## Introduction

Astroblastoma is a rare glial tumor of the central nervous system observed in both children and adults [1]. The incidence of astroblastoma constitutes about 0.45-2.8% of all neuroglial tumors [2]. These tumors are most commonly located in the cerebral hemispheres [3]. The prognosis of patients with astroblastomas depends on their grade; low-grade tumors have a favorable prognosis after gross total resection, while high-grade tumors have an uncertain prognosis [4]. Histopathological characteristics such as perivascular processes and vascular hyalinization can assist in identifying astroblastomas [4]. The investigation of choice for diagnosing astroblastoma is magnetic resonance imaging (MRI), where it appears as a large and lobulated tumor [4]. The origin of these tumors has been a subject of debate, as they exhibit similar histological features to ependymomas and astrocytomas [5]. Currently, gross total resection is generally regarded as the primary treatment approach, although chemotherapy and radiotherapy have demonstrated benefits, their exact roles remain undefined [6]. This case report addresses the diagnostic and therapeutic challenges of astroblastoma, a rare glial tumor of the central nervous system. By presenting a detailed case study of an 11-year-old boy with high-grade astroblastoma, the report aims to highlight the complexities in diagnosis and treatment. Emphasizing the importance of comprehensive clinical evaluation, advanced imaging techniques, and histopathological examination, the report contributes to the understanding of this rare tumor. Furthermore, it discusses evolving treatment modalities, including gross total resection and potential adjuvant therapies, underscoring the need for collaborative research efforts to improve outcomes for patients with astroblastoma.

## Case presentation

We present the case of an 11-year-old boy who visited the outpatient department (OPD) in October 2022 with left-sided mouth deviation, vomiting, and right-sided weakness over the past seven days. He had been in good health until one year ago when he started experiencing focal seizures involving his face. The patient was started on anti-epileptic medications which controlled his focal seizures but he had a generalized tonic-clonic seizure five months later. Since then, he remained seizure-free until the current presentation. Neurologically, the patient was alert, oriented, and cooperative, with a Glasgow Coma Scale (GCS) score of 15/15 and no cranial nerve deficits, sensory alterations, reflex abnormalities, or disturbances in higher mental functions. He had no reported allergies or significant family history, and his routine blood tests were normal.

**Figure 1:**
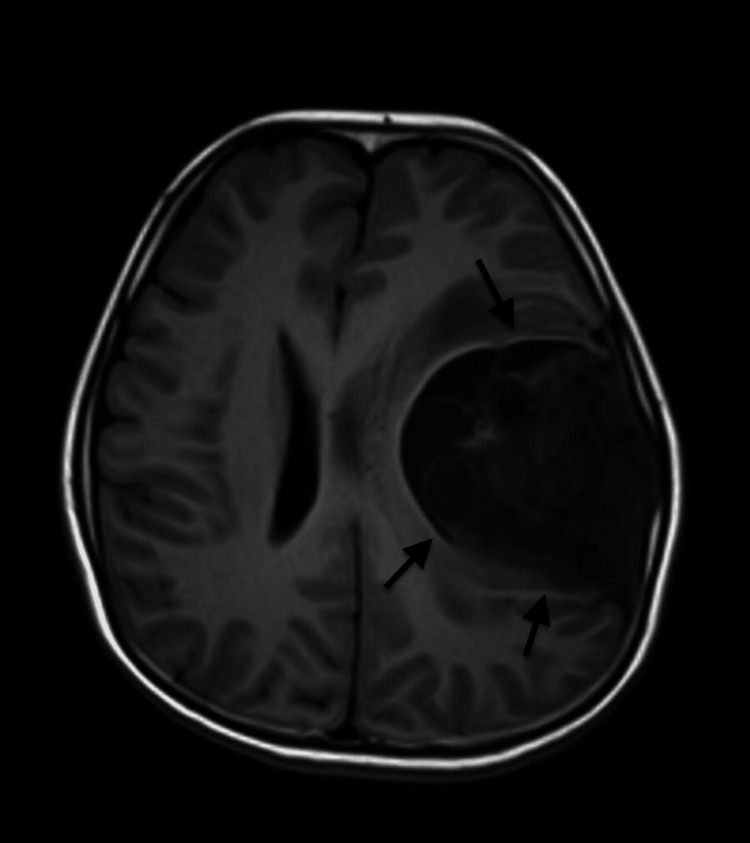
Preoperative magnetic resonance imaging scan in the T1 axial view Arrows are pointing toward the tumor.

**Figure 2:**
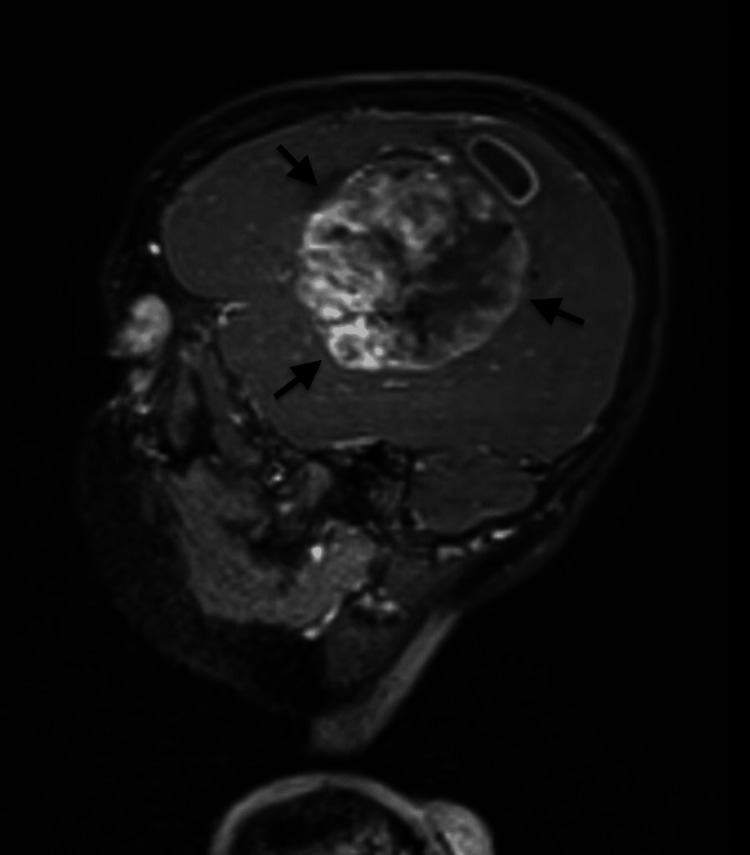
Preoperative magnetic resonance imaging scan in the T1 sagittal view Arrows are pointing toward the tumor.

**Figure 3:**
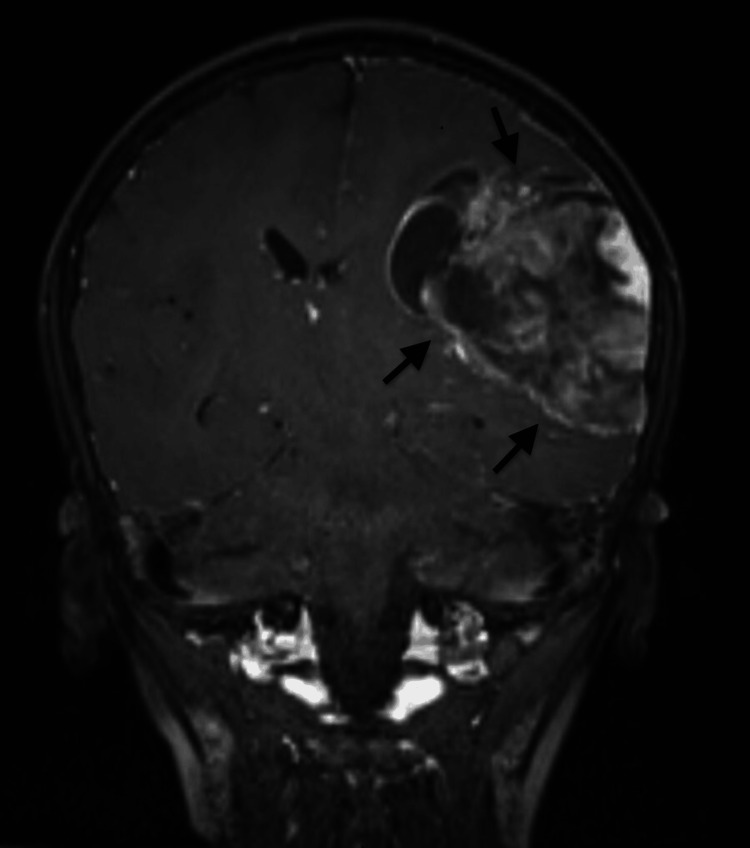
Preoperative magnetic resonance imaging scan in the T1 coronal view Arrows are pointing toward the tumor.

To determine the cause for his current signs and symptoms, an MRI of the brain with contrast was ordered which revealed a large solid cystic mass in the left parietal lobe, extending inferiorly into the left temporal lobe, measuring approximately 63 x 60 x 82 mm (AP x TR x CC). The mass appeared hypointense on TI and heterogeneous on T2 and fluid-attenuated inversion recovery (FLAIR) images. The solid component exhibited restricted diffusion in certain areas, resulting in mass effect and edema on the surrounding brain tissue. Additionally, there was an 8 mm midline shift to the right, along with compression of the ipsilateral ventricle. Heterogeneous post-contrast enhancement and mild adjacent dural enhancement were also observed. No mass was detected in the right cerebral hemisphere (Figures 1-3).

**Figure 4:**
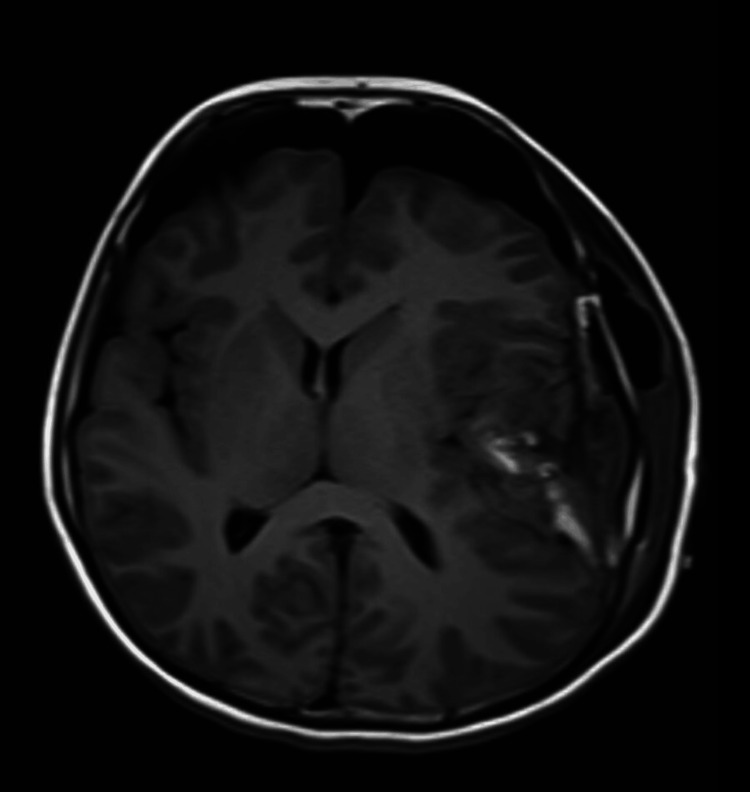
Postoperative magnetic resonance imaging scan in the T1 axial view This scan was performed three months after surgery and shows no visible enhancing lesion.

**Figure 5:**
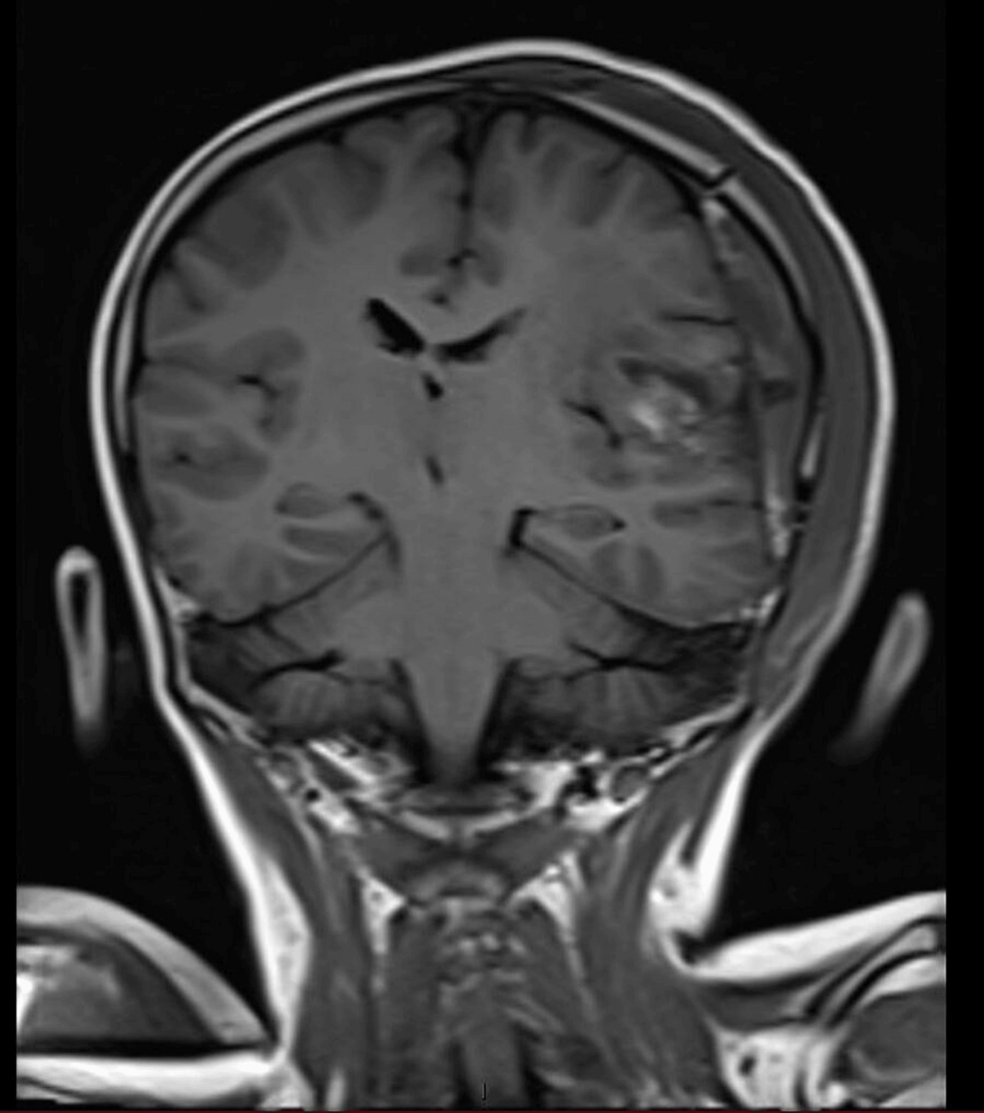
Postoperative magnetic resonance imaging scan in the T1 coronal view This scan does not show any residual tumor.

**Figure 6:**
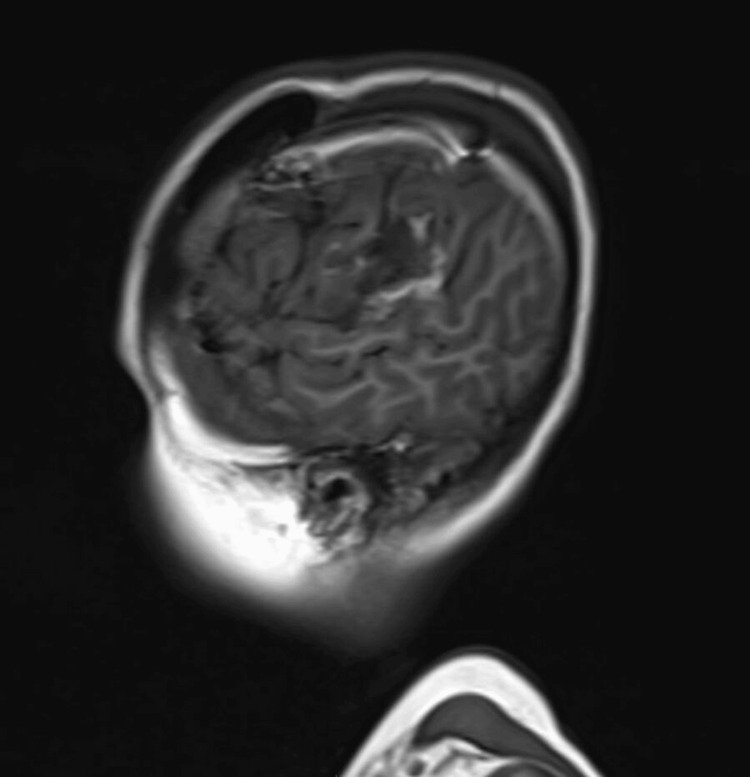
Postoperative magnetic resonance imaging scan in the T1 sagittal view The scan does not show any residual disease.

The patient was admitted the following day and underwent a left craniotomy for debulking of the lesion. The procedure was successful without any post-operative complications, achieving gross total resection. The tumor demonstrated high vascularity, with firm consistency in some areas and calcification in others. The vessels supplying the tumor were coagulated and excised to ensure hemostasis. Due to the association of a high-grade astroblastoma diagnosis with the likelihood of recurrence, tumor progression, and a worse prognosis, the patient underwent a follow-up three months post-surgery with an MRI of the brain. The results showed no tumor recurrence or any post-contrast enhancement indicating tumor growth (Figures 4-6).

**Figure 7:**
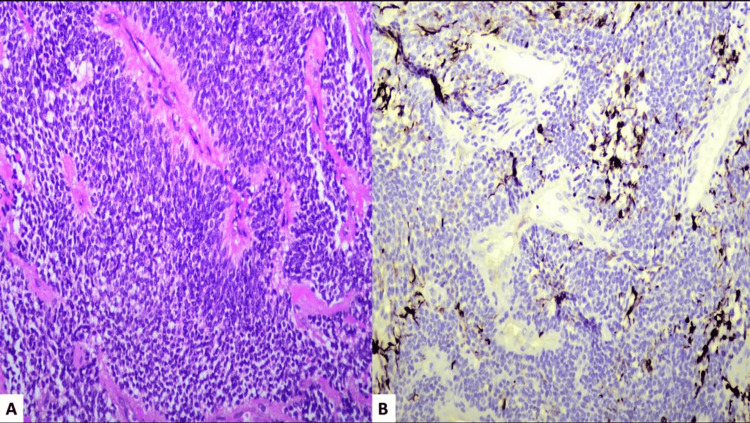
(A) Histopathology showing perivascular pseudorosettes and vascular hyalinization (H/E, 200x). (B) GFAP immunohistochemistry showing focal positivity in the tumor cells (IHC, 200x). H/E: Hematoxylin and eosin, IHC: Immunohistochemistry

Histopathological examination confirmed the diagnosis of an astroblastoma with high-grade features, including increased cellularity, high mitotic count, focal microvascular proliferation, and a high labeling index (Ki67). Immunostaining for GFAP, EMA, and D240 was positive. Perivascular pseudorosettes were observed throughout the tumor, and the nuclei appeared round to oval in shape (Figure 7).

After the surgery, the patient was transferred to the general floor. Although the patient exhibited normal mobility, there persisted right-sided weakness and left-sided mouth deviation. Nevertheless, the patient voided spontaneously, and approximately two days later, there was a slight improvement in his right-sided motor weakness, progressing from a flicker of contraction (Grade 1) to movement possible with gravity excluded (Grade 2). Additionally, the left-sided mouth deviation also slightly improved. Subsequently, on the following day, the patient was discharged with an oral pain management regimen, and a regular diet was also recommended.

At the follow-up visit, three months post-surgery, the patient had returned to his baseline. The patient's right-sided motor weakness was markedly improved from active movement with only gravity eliminated (Grade 2) to active movement against gravity with some resistance (Grade 4), and left-sided mouth deviation was not observed. The incision had healed very well with no signs of infection, such as pus. Additionally, the patient underwent radiotherapy with post-op MRI scans at three, six, and nine months, and there was no tumor regrowth observed. The patient continued to do very well without any motor weakness.

## Discussion

The clinical presentation of astroblastomas varies, with a prognosis falling between astrocytomas and glioblastomas; the diagnosis of astroblastomas is complex and prone to misdiagnosis, and radiopathological studies aid in distinguishing them from ependymomas [2]. On the contrary, ependymomas are much more common than astroblastoma and account for 6.8 percent of all glial tumors [7].

The frontal lobes supra-tentorial region is where the mass most frequently appears, followed by the parietal and temporal lobes [8]. The presentation of the case depends on the location and size of the tumor. Our patient had a history of epileptic seizures, but one of the most common presentations seen is headache, nausea, and vomiting secondary to raised intracranial pressure [2]. Studies have suggested that metabolic and structural changes within the tumor tissue affect the surrounding neuronal connections, manifesting as epileptic seizures [7].

Radiologically, our case had a typical large supra-tentorial mass that was solid and cystic. The location of the mass helped differentiate it from ependymomas, which are usually infratentorial and located within or adjacent to the ventricles [7]. Radiological studies also show calcification of the mass, which in our case was confirmed postoperatively. Oligodendroglioma can be differentiated from astroblastoma based on the type of calcification, which is punctate in astroblastomas and nodular in oligodendrogliomas [9-11]. On MRI it often has a solid and cystic component characterized by a bubbly appearance in the solid component [12].

Histologically, the presence of perivascular pseudorosettes, along with prominent vascular hyalinization that gives the characteristic cartwheel appearance, is typical for astroblastoma [12]. According to the latest WHO 2021 classification of central nervous system tumors, astroblastomas are defined as MN-1-altered circumscribed astrocytic gliomas, with MN-1 alteration present in approximately 70% of cases. In instances where MN-1 alteration is undetermined or absent, the tumor is characterized as not otherwise specified (NOS) or not elsewhere classified (NEC), and no formal grading has been assigned to it [13]. Since WHO has not assigned a formal grading, it is worth mentioning that Bonin and Rubinstein have proposed two distinct types based on histopathological presentation: high grade and low grade [14]. The low-grade type is more differentiated and benign-appearing, characterized by low mitotic activity, relatively less cellular atypia, minimal vascular endothelial proliferation, and sclerosis of the vascular walls, and has a better prognosis with surgical resection [14]. On the contrary, our case had increased cellularity and high mitotic rates, which are features of high-grade astroblastoma (anaplastic). While high-grade astroblastomas generally have a poor prognosis, favorable outcomes can be achieved in patients who undergo gross total resection of the tumor [2].

Immunostaining for astroblastomas is usually positive for GFAP and Vimentin, which support the theory that astroblastomas are derived from primitive astrocyte cell lines [12]. The cells also stain for S-100 and the results are variable for EMA, Cyto-keratin, CAM 5.2, and neuron-specific enolase (NSE). Immunostaining results can help differentiate astroblastomas from ependymomas. Ependymomas usually stain less intensely with GFAP [12].

Owing to the lack of data and the rarity of the disease, the optimal treatment of astroblastoma is still unclear. Currently, data suggests that gross surgical resection is the best option. Gross surgical resection is better than subtotal resection. Few single-center studies have reported better outcomes with the use of chemotherapy and radiotherapy post-operatively, though their role remains unclear and debatable [15]. A retrospective analysis of 239 patients with astroblastomas utilizing the Surveillance, Epidemiology, and End Results (SEER) program showed no significant difference in the prognosis of patients receiving radiotherapy before or after surgery. They found that patients with aggressive tumors were more likely to receive radiotherapy adjunct to surgery [16].

## Conclusions

Astroblastoma, a rare glial tumor of the brain, presents unique diagnostic and therapeutic challenges. Accurate diagnosis through advanced imaging and histopathological examination is crucial. While gross total resection remains the primary treatment approach, the role of adjuvant therapies, especially for high-grade tumors, is still evolving. Collaborative efforts and ongoing research are essential to improve outcomes for individuals with astroblastoma.
